# Animal Abuse: Helping Animals and People

**DOI:** 10.3390/ani3030606

**Published:** 2013-07-08

**Authors:** Eleonora Gullone

**Affiliations:** School of Psychology and Psychiatry, Monash University, Monash, VIC 3800, Australia; E-Mail: Eleonora.gullone@monash.edu

This six part book is edited by Catherine Tiplady from the Centre of Animal Welfare and Ethics at the University of Queensland, Australia. She has also authored most of the chapters and co-authored others. Other contributors include highly respected authorities such as Phil Arkow (the coordinator of the National Link Coalition) and Michael Byrne, QC (Barrister-at-law, Queensland Bar). 

**Part One** of the book comprises chapters examining definitions of animal abuse. All four chapters in this section are authored by Catherine Tiplady and cover: (i) the definition of animal abuse; (ii) the history of animal abuse; (iii) discussion of why people abuse animals; and (iv) discussion of why some people behave in a converse manner and care for animals. 

**Part Two** of the book comprises five chapters. Again, all five are authored by Tiplady and cover abuse across different areas and industries including animals used in the food and fibre industries, animals abused for entertainment including hunting, sport, and art. Other chapters examine philosophical, social, ethical and religious influences on our treatment of animals. I found this part of the book to include an enormous breadth of information, mostly touching the surface of most of the issues covered but certainly providing sufficient seed for the reader to pursue in depth elsewhere.

In **Part Three**, Phil Arkow discusses the *One Health* model which proposes the bridging of commonalities between human medicine and veterinary medicine. Through collaborative efforts among health science professionals, the objective of the One Health model is to work locally, nationally, and globally to optimize the health of people, domestic animals, wildlife, plants, and the environment. The proposed means of doing so is through building of closer interactions between different professional groups. Other chapters in this applied section of the book examine: (i) relationships between human and animal abuse; (ii) issues associated with sheltering abused animals and families together; (iii) A program that looks at fostering empathy in child victims of abuse through animal-assisted therapy. The final chapter in this section examines the very difficult topic of mental health issues arising from exposure to animal abuse during the course of one’s work and provides useful strategies with which to deal with such issues.

**Part Four **of the book explores ways of addressing animal abuse including chapters by other contributors on the behavioural consequences of animal abuse, for the animals. It also addresses ways of intervening to treat these adverse consequences. Discussion of prosecution of animal abuse and neglect is provided in Chapter 17. Tiplady authors the next two chapters which comprise a number of case studies and examination of humane euthanasia, respectively. 

**Part Five **contains chapters that focus on veterinary forensics and provide a wealth of useful advise for practicing veterinarians primarily with regard to the diagnosis of abuse.

The book ends with a final section (Part Six) made up of interviews with individuals who have played an influential role in dealing with societal abuse of animals. Their stories are both informative and inspirational.

Overall, this book has so much breadth in the area of animal abuse. The areas covered range from the very academic and philosophical right through to very applied areas including, among others, the diagnosis of animal abuse and interventions aimed at helping women who are victims of abuse to leave their situation without parting with their beloved companion animals. Given its breadth, it is relevant and recommended reading for many individuals including those who may or may not work with animals and who may or may not have companion animals as part of their family but who simply describe themselves as animal lovers or feel a sense of compassion or responsibility to treat sentient beings morally and with care. It is also relevant to those who work with human victims of abuse and those who work with animal victims of abuse. Above all, the book is educational as it provides a wealth of information regarding animal abuse across many different areas of society. Importantly, it also provides much information and inspiration about what caring individuals can do to help animal victims of abuse. 

